# Prevalence of Lhermitte's sign in multiple sclerosis versus neuromyelitis optica

**Published:** 2014

**Authors:** Masoud Etemadifar, Noushin Mehrbod, Leila Dehghani, Aryan Golabbakhsh, Mahboobeh Fereidan-Esfahani, Mojtaba Akbari, Zahra Nasr

**Affiliations:** 1 Department of Neurology, Isfahan Neurosciences Research Center, School of Medicine, Isfahan University of Medical Sciences, Isfahan, Iran; 2 Department of Neurology, School of Medicine, Isfahan University of Medical Sciences, Isfahan, Iran; 3 Department of Medical Sciences, School of Medicine, Islamic Azad University, Najafabad Branch, Isfahan, Iran; 4 Medical Students’ Research Center, School of Medicine, Isfahan University of Medical Sciences, Isfahan, Iran; 5 Department of Epidemiology, School of Medicine, Shiraz University of Medical Sciences, Shiraz, Iran

**Keywords:** Multiple Sclerosis, Neuromyelitis Optica, Lhermitt's Sign

Lhermitte's sign (LS) is one of the sensory injuries of the spinal cord which is frequent in some demyelinating diseases. The sign refers to a transient electric-shock like sensation and sometimes tingling or buzzing in the neck that runs down the spine and into limbs. It is triggered by movement including the flexion and rarely by extension or rotation of the neck, walking and bending and even sometimes it is spontaneous and can be attend by a sense of intend pain.^1, 2^ Although LS is not limited to multiple sclerosis (MS), it is highly prevalent in MS and other similar demyelinating disorders e.g. neuromyelitis optica (NMO).^3^ This cross-sectional study was conducted in Isfahan MS Society (IMSS) from April 2003 to July 2010. This study aimed to inspect the prevalence of LS among MS and NMO patients. All the patients were asked for the history of LS at presentation or during subsequent clinical surveillance. LS was considered to be positive if a transient electric shock sensation, tingling, rippling or other feelings had travelled rapidly along the neck and limbs.^3^ If the symptom had occurred, details of clinical and demographic features were determined based on follow-up records. The study protocol was approved by institutional Ethics Committee. A total of 3522 MS patients and 78 NMO patients were enrolled in this study. 153 patients with MS (including 41 males and 112 females) and 16 patients with NMO (including 8 males and 8 females) with positive history of LS were recruited. At the time of interview, their ages were ranged from 15-50 years, with mean age of 29.26 ± 7.26 in MS patients and 28.31 ± 6.98 years in NMO patients. The prevalence of LS among MS patients (4.3%) was significantly lower than NMO patients (20.5%) (P < 0.0001). 5.9% of the MS and 12.5% of the NMO patients had a positive family history of Lhermitte's sign. It was observed that a higher proportion of patients with NMO rather than MS experienced the sign (20.5% vs. 4.3%). This is comparable to a study by Kanchandani in which has been reported that 33.3% of 114 patients with MS experienced LS.^1^ It is also noteworthy that in the past NMO was considered as one of the subtypes of MS which can justify that maybe these diversities are due to the fact that in the past, LS positive patients with NMO, were reported as LS positive patients with MS. Although the precise etiology of LS is still elusive, previous studies reveaed that movement of demyelinated axons in posterior columns of the spinal cord could be a rational explanation.^3^ According to previous studies, patients presenting with LS may also have some signs of vitamin B^12^ deficiency.^2^ Deficiency of vitamin B^12^ (Cobalamin) is a potential cause of neurologic complications such as demyelination of axonal sheath in central and peripheral nerves which manifests with general weakness and paresthesia and sometimes LS. In line with these manifestations, vitamin B^12^ deficiency may serve as a metabolic factor for imperfect methylation of a major component of CNS myelin which is myelin basic protein (MBP). Several studies demonstrated low levels of vitamin B^12^ in MS patients but there is still no report about the level of the Cobalamin in NMO patients.^4^ Hence, a question that arises from the preceding data is, whether there is any association between Cobalamin levels and occurrence of LS in patients with MS and NMO. In line with other studies we observed that cervical plaques are more evident among NMO patients and also a higher proportion of patients with NMO experienced LS rather than MS patients.^5^ Overall, there is a higher prevalence of LS among NMO patients than MS patients. Further studies should be performed in order to support our findings.
